# Meniscal allograft transplantation: a meta-analysis

**DOI:** 10.1051/sicotj/2017016

**Published:** 2017-04-21

**Authors:** Manolito De Bruycker, Peter. C.M. Verdonk, René C. Verdonk

**Affiliations:** 1 Faculty of Medicine, Ghent University De Pintelaan 185 B9000 Gent Belgium; 2 Antwerp Orthopaedic Center, AZ Monica Hospitals Harmoniestraat 68 B2018 Antwerp Belgium; 3 Department of Orthopaedic Surgery, Antwerp University Hospital Wilrijkstraat 2650 Edegem Belgium; 4 Department of Orthopaedic Surgery, Campus Erasme, University Libre de Bruxelles Route de Lennik 808 1070 Anderlecht Belgium

**Keywords:** Knee, Meniscal allograft transplantation, Outcome

## Abstract

*Purpose*: This meta-analysis evaluates the mid- to long-term survival outcome of MAT (meniscal allograft transplantation). Potential prognosticators, with particular focus on chondral status and age of the patient at the time of transplantation, were also analysed.

*Study design*: Meta-analysis.

*Methods*: An online database search was performed using following search string: “meniscal allograft transplantation” and “outcome”. A total of 65 articles were analysed for a total of 3157 performed MAT with a mean follow-up of 5.4 years. Subjective and clinical data was analysed.

*Results*: The subjective and objective results of 2977 patients (3157 allografts) were analysed; 70% were male, 30% were female. Thirty-eight percent received an isolated MAT. All other patients underwent at least one concomitant procedure. Lysholm, Knee injury and Osteoarthritis Outcome (KOOS), International Knee Documentation Committee (IKDC) and Visual Analogue Scale (VAS) scores were analysed. All scores showed a good patient satisfaction at long-term follow-up. The mean overall survival rate was 80.9%. Complication rates were comparable to standard meniscal repair surgery. There was a degenerative evolution in osteoarthritis with at least one grade in 1760 radiographically analysed patients. Concomitant procedures seem to have no effect on the outcome. Age at transplantation is a negative prognosticator. The body mass index (BMI) of the patient shows a slightly negative correlation with the outcome of MAT.

*Conclusions*: MAT is a viable solution for the younger patient with chronic pain in the meniscectomised knee joint. The complications are not severe and comparable to meniscal repair. The overall failure rate at final follow-up is acceptable and the allograft heals well in most cases, but MAT cannot be seen as a definitive solution for post-meniscectomy pain. The correct approach to the chronic painful total meniscectomised knee joint thus requires consideration of all pathologies including alignment, stability, meniscal abnormality and cartilage degeneration. It requires possibly combined but appropriate action in that order.

## Introduction

The meniscus is known to have an important role in distributing load, enhancing stability, facilitating congruency and contributing to lubrication and nutrition in the knee. Clinically and scientifically there is evidence that a meniscal deficiency can attribute to the development of premature osteoarthritis. Total meniscectomy increases the peak contact stresses in the affected compartment, accounting for an estimated 4% cartilage loss per annum (greater on the lateral side) [1]. The amount of removed meniscal tissue attributes to this increase in contact stress. Meniscus allograft transplantation is most commonly indicated in the symptomatic patient who is meniscus deficient. In particular, it is most suitable for patients under the age of 50 years with debilitating pain localized to the tibiofemoral articulation but who still have not developed advanced degenerative changes in the knee. It may also be indicated in patients undergoing reconstruction of the anterior cruciate ligament (where there is concomitant meniscal deficiency and a resultant risk of excessive forces acting through the reconstructed ligament). Meniscal allograft transplantation (MAT) may also be considered as a prophylactic chondroprotective procedure in the young but still asymptomatic patient with complete meniscal (mostly lateral after discoid meniscal resection) deficiency. While there is little evidence to directly support this third indication at present, long-term outcome data for the procedure may aid further discussion of its potential role here. Current intermediate term data demonstrates that meniscus allograft transplantation yields good to excellent results in terms of pain, function and activity levels in up to 85% of patients at up to eight years following transplantation [2, 3]. The goal of this meta-analysis was to provide clinical and radiological outcome data, in addition to the survival analysis of meniscus allograft transplantation. A secondary goal was to identify prognosticators that may influence this process, with particular focus on chondral status and age of the patient at the time of transplantation. As it is stated that a meniscal allograft transplantation can be a solution for post-meniscectomy pain, our hypothesis is that there should be a difference in subjective results before index surgery and at final follow-up. As for the clinical data it was hypothesized that there should be little to no difference in outcome between short-term and long-term results for MAT to be a viable solution.

## Materials and methods

**Figure 1.**
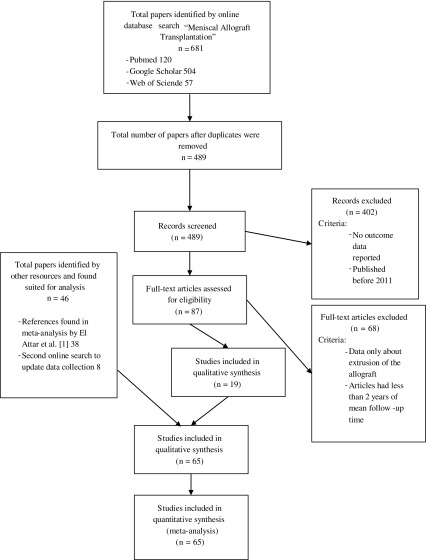
Prisma study flowchart.

From 2015 to 2016 the databases PubMed, Web of Science and Google Scholar were searched using the term “Meniscal Allograft Transplantation”. An initial search resulted in 120 articles in PubMed, 57 in Web of Science and 504 in Google Scholar. The term “outcome” was added as it was the scope of this study to evaluate the outcome after MAT. This search was further refined by only searching for studies published in English in the last five years. This eventually gave a total of 87 articles, which were meticulously reviewed. It was then decided to exclude all articles that reported results with a follow-up of less than two years. Articles only containing data about meniscal extrusion were also excluded as this was not the main scope of the study. This delivered a total of 19 articles published from 2011 until 2016. To get an overview of the outcome after meniscal allograft transplantation in a time span of 32 years, all articles analysed by El Attar et al. were also included [2]. In July 2016, it was decided to perform a new search with the same variables to update our data pool with the most recently published data. This led to the inclusion of a further eight articles (Table 1). This means the total number of studies used in this analysis is 65, of which 36 were prospective cohort studies or case series and 29 were retrospective studies. Eight authors published studies from the same patient pool, but at different follow-up times [3–19] (Figure 1). Statistical analysis was performed using IBM^®^ SPPS^®^ Statistics 21. A confidence interval of 0.05 was used for all analyses. Initial data collection was done with Microsoft^®^ Excel 2016. Tables were also computed with Excel. Cases with the same values were grouped in all graphs. Data reported in all these articles accounts for 3157 allografts in 2977 patients. Subjective and clinical outcomes were analysed. In case of the subjective findings only the results of the most used questionnaires were analysed, being Lysholm-, KOOS-, IKDC- and VAS-scores. Pre-operative scores were compared with scores at final follow-up for all of these questionnaires. For all questionnaires the hypothesis was the same: there should be a significant difference between the pre-operative scores and the scores at final follow-up if a MAT is a viable solution for post-meniscectomy pain. A Wilcoxon signed ranked test was used for these comparisons. Clinical survival rate was analysed using the Kruskal-Wallis test with mean time of follow-up as a grouping variable. As hypothesis, it was stated that there should be little to no difference between results of short- and long-term follow-up. A one-way analysis of variance (ANOVA) test was performed to define if there was a difference in outcome after MAT using different surgical techniques and differently preserved menisci. Complications, arthroscopic and radiologic findings were grouped and overall findings were stated, however they were not statistically analysed due to heterogeneity of these findings. Potential prognosticators (age, sex, BMI, number of concomitant procedures) were also investigated to search for a possible correlation between these prognosticators and the outcome of MAT. To evaluate these data, articles were grouped together.

**Table 1.** Patient selection criteria used by the authors.

Inclusion criteria	Exclusion criteria	
Age under 45, 50 or 55	Osteoarthritis	Rheumatoid arthritis
Normal alignment of the joint	Axial malalignment	Neurologic disease
No ligament surgery	Instability of the knee	Pregnancy
Post-meniscectomy pain and swelling during ADL, sport	Arthrofibrosis	Osteonecrosis
Stabilized AC (before or associated to MAT)	Muscle atrophy	Osteophytosis
Meniscal tissue loss of more than 50%	Joint infection	>Ahlback II on Rosenberg RX
OT possible associated with MAT	Synovitis	Immature bones
	PC surgery	Age above 60
	Outerbridge class III or higher	Severe cartilage degeneration
	Corticosteroids less than 30 days before transplant	

ADL: activities of daily living, ACL: anterior cruciate ligament, MAT: meniscus allograft transplantation, OT: osteotomy, PCL: posterior cruciate ligament.

## Results

### Patient demographics

All articles together provided us with the results of 2977 patients of which 1982 were male, 898 were female and 97 were undefined. The mean age ranged between 9 and 51 years, with an overall mean of 33 years. The youngest patient recorded was one year old, the oldest was 73 years old. A total of 3157 allografts were analysed. There were 1594 lateral meniscal transplantations, 1451 medial meniscal transplantations, 59 combined and 53 were undefined. The mean follow-up time ranged from half a year to 15 years with an overall mean follow-up of 5.4 years. The shortest follow-up time was only two months and the longest follow-up time was 25 years. Patients mostly underwent the allograft transplantation at a mean time of 10 years post-meniscectomy. Chondromalacia gradation was reported for 1059 patients. In this population, 56% had osteoarthritis, 42% had minimal osteoarthritis and 2% had no chondral damage (based on Claes et al.) [3–70]. The Coleman Methodology Score (CMS) and Modified Coleman Methodology Score (MCMS) according to Kon et al. were calculated for all articles. The mean CMS is 61.8 (range 25–80) and the mean MCMS is 52.7 (range 24–69) [71–72].

### Patient selection

To define the population suited for a meniscal allograft transplantation many different variables are used. Surgery is usually indicated in patients with chronic post-meniscectomy pain, swelling and functional disability of the knee. Most authors only include patients with a normally aligned and stable knee. Patient’s age is also limited to 50 years in most investigations. Contra-indications involve severe osteoarthritis, malalignment, arthrofibrosis, muscle atrophy, infection, synovitis, neurologic disease, osteophytosis, osteonecrosis, immature bones, immunologic disorders such as diabetes and rheumatoid arthritis. Morbid obesity is also seen as a contra-indication (Table 1) [3–19, 21–70].

### Graft selection and fixation technique

In order to have a proper fitting allograft plain radiograph, computed tomography (CT) and magnetic resonance imaging (MRI) were performed routinely. In the early studies, height and weight matches were standard. In the early studies, such as those performed by Wirth et al. and Verdonk et al., allograft transplantation was performed with an open approach. All the other studies report the outcome after arthroscopically assisted MAT [3–19, 21–70] (for a full overview, see Appendix A). As for allograft preservation various techniques are used. For a total of 2592 patients or 2853 allografts the used technique was clearly described. In 385 cases the preservation technique was not defined or unclear (Table 2). It becomes clear a deep-frozen or cryopreserved technique is preferably used because of several practical advantages. The allograft is better preserved (although it loses its viability) and because of less manipulation infection is greatly avoided. However, there is no significant difference found in allograft survival at final follow-up after a one-way ANOVA (*P =* 0.086) [3–19, 21–70].

**Table 2.** Overview of preservation techniques used.

Preservation technique of the allograft	Amount of allografts	%
Deep-frozen	1335	42.3
Cryopreservation	768	24.3
Viable	368	11.7
Irradiated	1	0.0
Lyophilized	17	0.5
Nondefined/Not specified	668	21.2
Total	3157	100

In this meta-analysis, the used allograft fixation technique was described for all allografts. The most commonly used fixation technique was a bony fixation (37.1%), followed by a soft tissue fixation (34.7%) and a tunnel fixation (18.8%). For a total of 9.4% of this population the fixation technique was not explicitly described. (Table 3) Analysis of clinical data using a one-way ANOVA test did not show a significant difference in outcome after transplantation between these groups (*P* = 0.419) [3–19, 21–70].

**Table 3.** Fixation technique.

Fixation technique	Amount of allografts	%
Bony	1171	37.1
Soft tissue	1094	34.7
Transosseous	595	18.8
Nondefined	297	9.4
Total	3157	100

**Table 4.** Osteoarthritis grading of available patients^a^.

	*n*	%
Pre-operative grade of osteoarthritis		
Normal cartilage	229	38.4
Minimal osteoarthritis	174	29.2
Osteoarthritis	193	32.4
Total	596	100
Post-operative grade osteoarthritis		
Normal cartilage	100	25.5
Minimal osteoarthritis	156	39.8
Osteoarthritis	136	34.7
Total	392	100

a All patients eligible for grading were selected and regrouped based on the classification published by Claes et al. [20].

### Associated procedures

**Figure 2.**
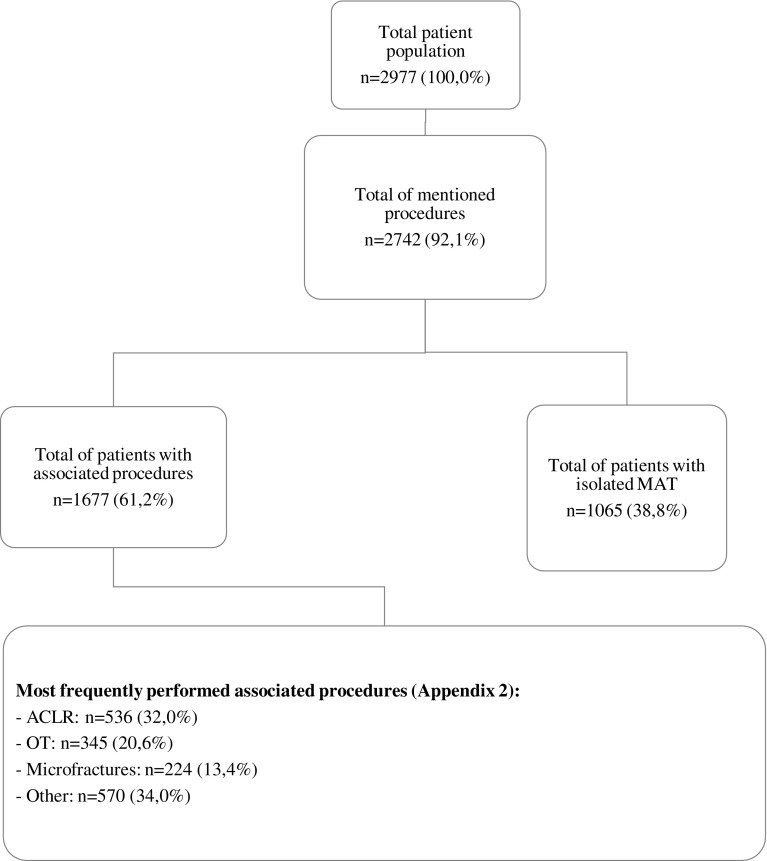
Overview of associated procedures. As shown only 38.8% of the patient population underwent isolated MAT. Most frequently an anterior cruciate ligament reconstruction was performed, followed by an osteotomy of the fibula or tibia. Other procedures performed were all done in attempt to optimize the chondral surface and the alignment of the knee.

Information on associated procedures was available for 2742 patients (92.1%). Only 39.1% of this population received an isolated MAT. In all other cases, the patient was subject to associated procedures. Most frequently a concomitant anterior cruciate ligament reconstruction (ACLR) or corrective osteotomy was performed. Other procedures were related to optimizing the chondral surface (Figure 2; see Appendix B) [3–19, 21–39, 41–68].

### Outcome

#### Subjective findings

**Figure 3.**
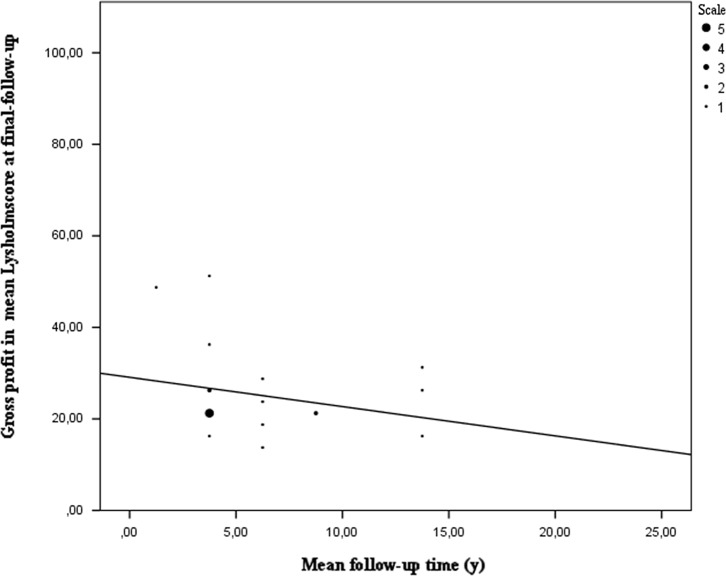
Gross profit in mean Lysholm score based on mean follow-up time. At short- to mid-term follow up an overall profit of at least 20 points in Lysholm score can be seen which means patients feel a significant improvement in their daily functioning. However later on they tend to have a lesser improvement as a decline in gross profit can be observed at a longer follow-up time.

Using the Lysholm-, VAS (visual analogue scale)-, IKDC (international knee documentation committee)- and KOOS (Knee injury and Osteoarthritis Outcome)-scores, the patient’s subjective evaluation after MAT was analysed. A total of 840 patients (28.2% of the total population) filled in the Lysholm questionnaire before index surgery and at final follow-up. These questionnaires were uniform in all studies that reported these results. All patients gain a significant profit after MAT with a mean profit of 25% (7%–49%). Using a Wilcoxon signed rank test to compare pre-operative Lysholm scores and Lysholm scores at final follow-up showed that there is a significant difference between these scores (*P* < 0.005). However, it can be noted as shown in Figure 3 that patients who filled in the Lysholm questionnaire report less profit of MAT [5, 9, 11, 14, 17, 21, 23, 29, 40, 45, 46, 54, 57, 61, 62, 64, 66].

**Figure 4.**
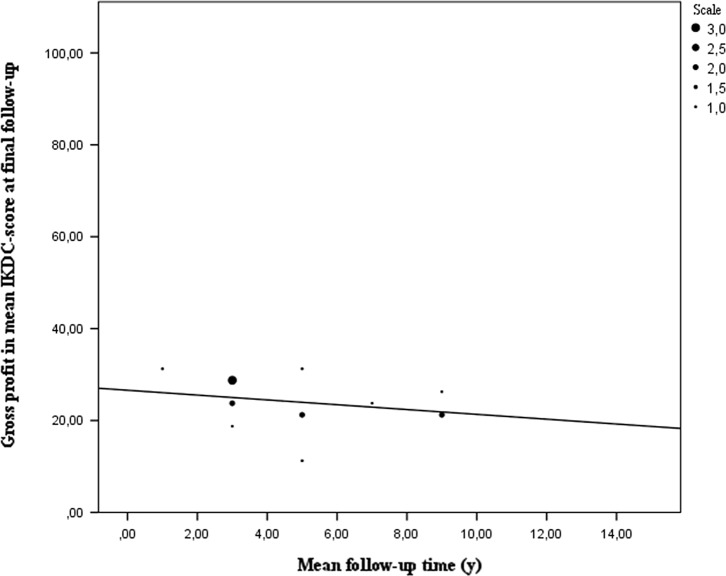
Gross profit in mean IKDC-score based on mean follow-up time. The gross profit in IKDC-score is on average 24 points. The biggest improvement in daily living can be observed in patients interviewed at short-to mid-term follow-up. A declining trend in the IKDC-score over time is also present.

The IKDC-scores from 442 patients (14.8% of the total population) also show a significant gain in quality of life and functionality according to the patients after performing a Wilcoxon signed rank test. (*P* = 0.001) There is a mean profit of 24 points for all 442 patients [4–23]. Patients who filled in this questionnaire at a longer follow-up time tend to have less profit of MAT (Figure 4).

**Figure 5.**
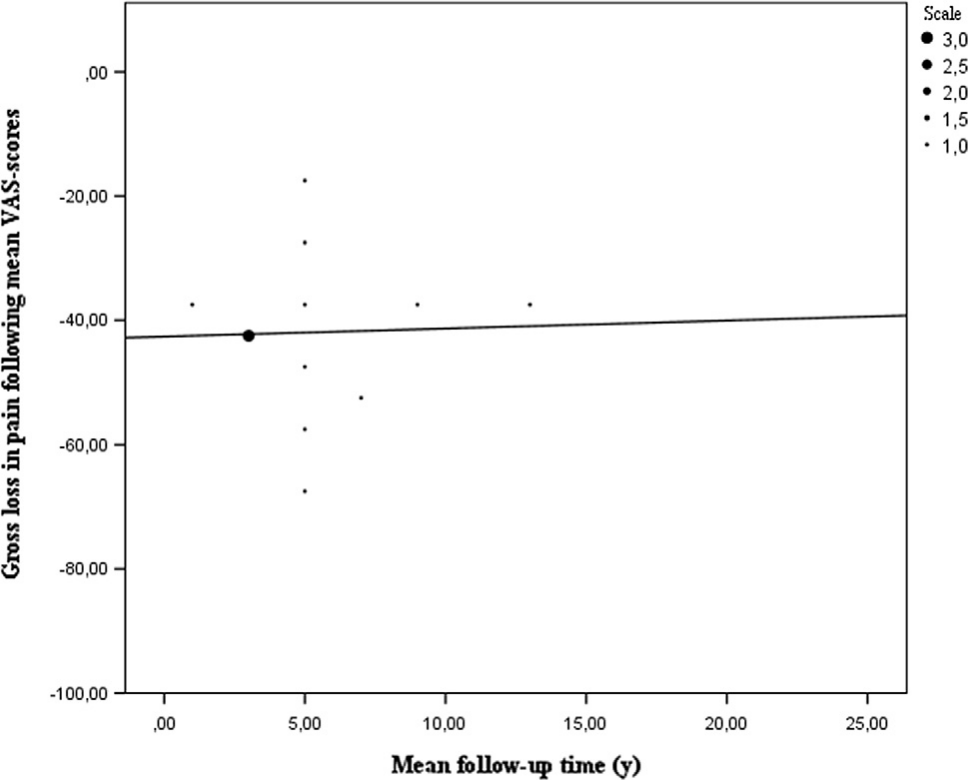
Gross loss in mean VAS pain-score based on mean follow-up time. Patients tend to have less pain after MAT as shown in Figure 4. On average the analysed articles reported a loss of pain of around 40 points on the VAS-scale at short- to mid-term follow-up. Articles reporting VAS-scores at longer follow-up times reported an increase in pain, thus their patients reported less loss of pain.

**Figure 6.**
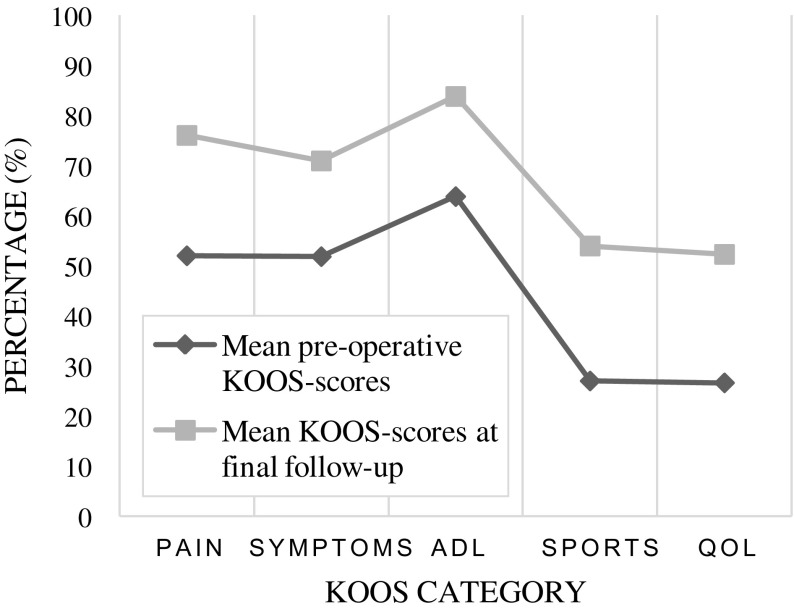
Difference in mean pre-operative KOOS-scores and mean KOOS-scores at final follow-up. In all categories of the KOOS-questionnaire there is a difference in scores of about 20%. All articles reporting KOOS-scores see an improvement in all categories at final follow-up. As shown sport activities and quality of life are scored as being acceptable at final follow-up, which is important as they tend to state that their quality of life is below average before index surgery.

Furthermore, the VAS- and KOOS-scores were also analysed, respectively, represented by 463 and 406 patients out of 2977. For both scores again a Wilcoxon signed rank test was used to analyse the data pre-operative and at final follow-up. The VAS-score shows that patients feel an improvement after MAT (*P =* 0.001). Less pain and swelling of the knee joint allow for more of their daily activities. However, pain seems to return over time, as patients who filled in the VAS-chart tend to report more pain (Figure 5) [4, 5, 15, 17, 21, 22, 27, 33–35, 37, 38, 47]. In the analysis of the KOOS-score, 12 papers were included. In all of the five categories questioned in the KOOS-questionnaire, there is a significant difference in mean scores between the pre-operative evaluation and evaluation at final follow-up (*P* < 0.001). As shown in Figure 6, there is a mean increase of 23.1 points at final follow-up. As the mean follow-up is six years for all these patients, the conclusion is that at a mid-term outcome patients experience a significant increase in their functionality after MAT [4, 5, 40, 48, 53, 54, 57].

In conclusion, the majority of patients experience a significant improvement in their daily living activities as in their sporting activities. At all times, being it a short-, mid-term or long-term outcome, the overall patient satisfaction is good. Although all of these subjective scoring questionnaires show a significant improvement, there is a declining trend in scores over time [3–11, 13–17, 19, 21–23, 25–54].

#### Clinical findings

**Figure 7.**
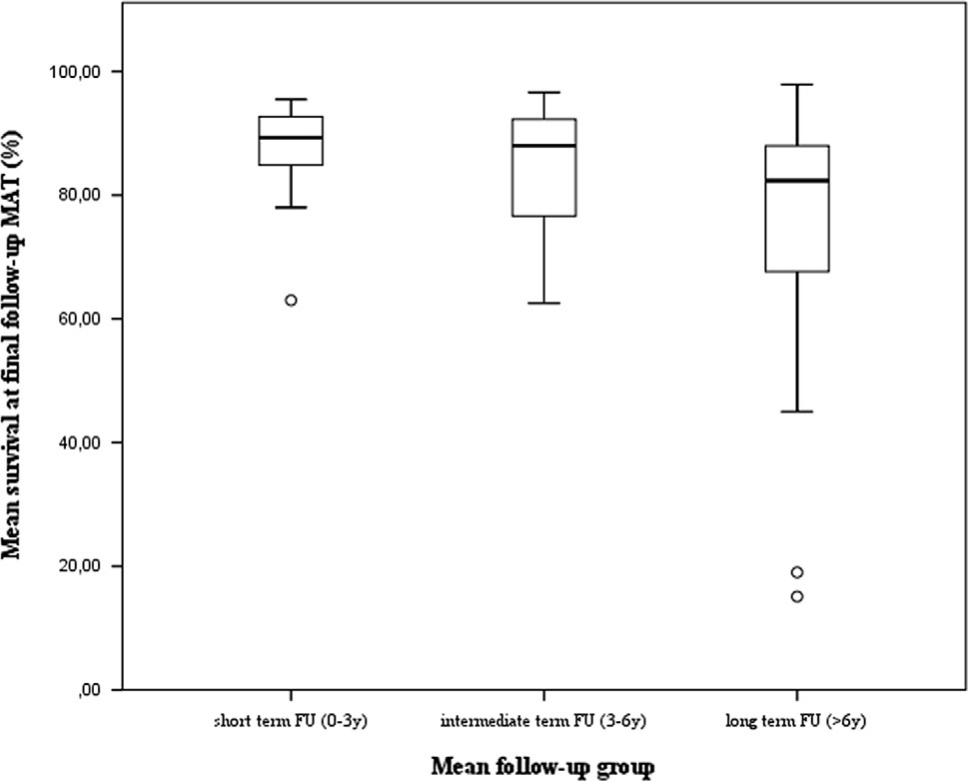
Mean survival at final follow-up defined by three mean follow-up groups. It can be observed that data at long term follow-up has a bigger interval. Thus it seems there’s a heterogeneity among these studies. Indeed it could be observed that in this group articles who used subjective problems such as pain tend to report a lower mean survival rate. Furthermore studies with a follow-up of more than 20 years are depicted in the two outliers. This indicates that more research has to be done to evaluate the lifetime of a meniscal allograft using more uniform failure criteria.

**Figure 8.**
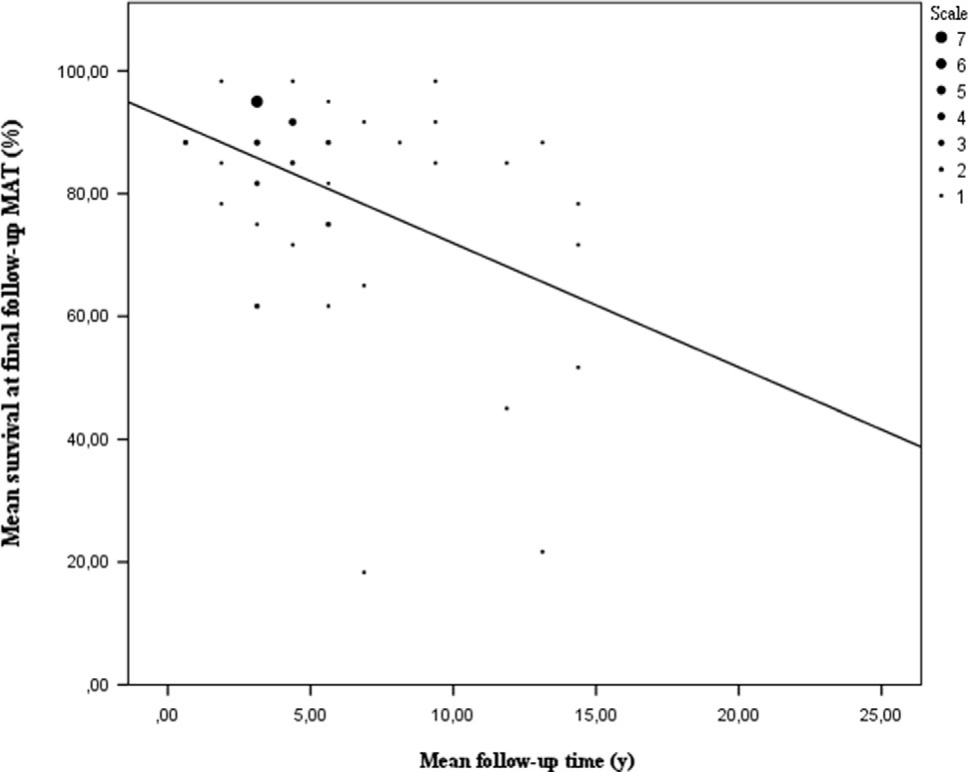
Mean survival at final follow-up by mean follow-up time. It is clearly depicted that the mean survival rate of the allograft linearly declines with follow-up time. As depicted at a mean follow-up time of 10–15 years almost half of the allografts tend to fail. The heterogeneous spreading of data at this mean follow-up time can be ascribed to the heterogenity of failure criteria used. The same can be stated for the outlier at a mean follow-up of seven years. Figure 7 also depicts tthat most of the data collected lies between a mean follow-up of 2–10 years.

The surgical technique was analysed first. In total, 53 of 65 studies investigated survival of MAT. This included 2677 patients representing 2835 transplants. The mean survival rate for these allografts was 80.9% (15.1%–97.9%). To further investigate the impact of time since surgery three mean follow-up categories were studied: <3 years, 3–6 years, >6 years. After analysis using a one-way ANOVA test, there seems to be a significant difference between these follow-up groups (*P* = 0.021). A further paired analysis of these groups using a Kruskal-Wallis non-parametric test showed no difference in outcome can be found between the mean short-term and mean mid-term follow-up group (*P* = 0.435). There is also no difference in outcome between mid-term and long-term follow-up groups (*P* = 0.074). However, a difference in outcome can be found between the mean short-term and mean long-term follow-up group (*P* = 0.04). There is a difference of 14.8% between these two groups in mean survival rate (Figure 7). These results need to be interpreted with care. Most of the data are collected at a mean short- to mid-term follow-up (Figure 8). It also shows outliers in mean mid-term to mean long-term follow-up survival data, which could have affected the results. These outliers were authors who used multiple endpoints as criteria for failure such as reoperation, extrusion, meniscal tears, pain and alternate findings after imaging. It is also important to point out that studies with a follow-up time of more than seven years report greater failure rates [6, 8, 13, 37, 40, 51, 65, 66]. Most authors define failure as a clinical failure, being it a meniscectomy, a graft repair or an arthroplasty with or without a prosthesis. Some of them use more strict failure criteria such as structural damage, alternate imaging, pain or reoperation not related to the graft. As a result these authors report higher failure rates. These findings make it difficult to draw a definite conclusion, thus a more homogeneous approach of reporting survival data is needed. A total of 167 out of 1665 patients converted to a prosthesis at a mean time of 10.5 years [3–8, 12, 13, 16–19, 23–27, 30, 33, 39, 53, 62, 65]. The complication rate of MAT is comparable to standard meniscal repair surgery. Based on a subpopulation of 1497 patients there is a complication rate of 15.6% (2.0%–51.4%) or 663 reported complications. A meniscal tear of the allograft is the most frequent complication, followed by debris removal in the joint space [5, 7, 9–16, 18, 19, 21–27, 29–31, 34–40, 42, 44, 45, 47, 50–58] (see Appendix C for a complete overview of all complications reported).

#### Arthroscopic findings

At index surgery for MAT a total of 999 knees were evaluated for chondral damage. Most of the authors report a definite change in the chondral surface, even though they used different selection criteria which excluded patients with high chondral wear. The pre-operative evaluation of the cartilage using MRI-imaging seems to be just an indication of possible osteoarthritis, but not a definite marker of the actual state of the cartilage. A total of 459 knees had moderate to severe osteoarthritis, so they did not meet the indication. Only a limited number of authors found no significant difference in survival rate, thus the osteoarthritis grade should still be considered pre-operatively [7, 9–19, 21–23, 27–30, 34–37, 39, 42–49, 53–59, 67, 69].

A post-operative arthroscopy was performed in 932 cases. Mostly because of a complication, only 479 patients had a post-operative arthroscopy for the sole purpose of evaluating the allograft and chondral surface. An important finding is that most of the patients had only a slight progression of osteoarthritis with a mean of one grade [20]. This restrains the chondroprotective effect suggested by many authors in long-term follow-up [7, 9, 11–19, 21–26, 29–32, 34–36, 38, 41–48, 51–58].

When evaluating the allograft most of the authors reported a good integration and healing of the allograft, which can also be seen histologically. Seven studies report the presence of shrinking of the meniscus allograft at evaluation. However, this is a difficult variable to analyse [3, 7, 11, 15, 26–31, 33, 35–37, 48, 54, 57].

#### Radiologic findings

A total of 1760 patients were available for analysis. Most of the patients were evaluated with a standing anteroposterior RX or with a Rosenberg view combined with MRI. To make a thorough analysis possible the best way to combine all the different classifications was used. Therefore it was chosen to merge all these classifications using the classification system introduced by Claes et al. [20]. This way pre- and post-operative osteoarthritis grades could be evaluated in a more coherent way. In Table 4, all available patients are classified into specific osteoarthritis groups. This confirms the findings found by the authors during arthroscopy. In all classifications, an overall decrease of one grade was found. These findings indicate that there is no long-term chondroprotective effect of MAT, but that the progression of osteoarthritis is delayed by a statistical mean of 10.5 years [6–9, 12, 17, 21, 22, 24, 35–38, 40, 43, 46, 51, 55–57, 60, 63, 67].

Some authors retain a narrowing of the joint in weight-bearing evaluations (327 patients). Overall the authors report no significant joint space narrowing, but in most cases there is some narrowing. But when compared to the contralateral knee, the difference is not significant. These results indicate that MAT provides a good function and integration in the index knee [3, 7, 29, 31, 33, 36, 37, 40, 43, 52, 53].

Furthermore, a total of 627 allografts were graded with MRI. Three grades were withheld: Grade 1: minimal hyper intensity in a specific meniscal area. Grade 2: a linear abnormal intensity in the complete allograft, divided into two subgroups (a) linear abnormal intensity; (b) linear abnormal intensity with a single image expansion and (c) globular-wedge-shaped hyper intensity. Grade 3: completely abnormal hyper intensity expanded to at least one joint surface area. About 75% of these allografts had a grade one or two. However, this does not implicate that these allograft were failures. Most of them were abnormal on MRI, while they were clinically asymptomatic. These allografts also had some structural changes. Most of the time they healed well but with some minor effusion. In some cases, there is also some minor displacement of the allograft, depicted clinically by complaints of limited knee joint mobility. In most of these cases, the procedure has failed as the displacement is a result of loose stitches or bone plugs. Shrinking also occurs. Usually 25% or sometimes up to 50% although this is difficult to measure [3, 7, 29, 31, 33, 36, 37, 40, 43, 52, 53].

#### Prognosticators

The overall mean age was 34 years. After a Spearman correlation analysis it was found that age is no determinant variable for the outcome at final follow-up after MAT, possibly because of the use of mean age (*P* = 0.795). However, individual studies report age-related outcomes [3–19, 21–70].

Van der Wal et al. found a difference in outcome between men and women. But it was observed only in the subjective questionnaires. Other authors did not find this difference. As results were not reported individually for men and women, an analysis could not be performed in this study [3–19, 21–70].

In total, five studies also evaluated the effect of the body mass index (BMI) on outcome after MAT. Only Saltzman et al. and McCormick et al. reported significant changes. They reported a minor negative effect on outcome after MAT when the patient’s BMI was higher than 25. The other three authors did not find any significant difference [4, 5, 44, 47, 48, 57].

A possible effect of concomitant procedures on the outcome after MAT was also considered. It appears there is no major difference between isolated MAT and MAT concomitant with other procedures. Only a small group of authors mentioned the effect on outcome. As all these data were already synthesized in all articles, a statistical analysis could not be done. However, most authors report they did not see a correlation between the number of procedures concomitantly performed with MAT and the outcome after MAT. Possibly because of the fact that these procedures are done because they stabilize the knee joint and optimize the chondral surface, which means the allograft can integrate properly. Furthermore, the time to transplant also does not have an influence on the outcome after MAT following a Mann-Whitney *U* test comparing the outcome between a short and long time to transplant (*P* = 0.445) [3–19, 21–70]. It is important to note that all these prognosticators were analysed based on means, thus it is certainly not conclusive and needs more research.

## Discussion

This meta-analysis included 2977 patients, 1982 males and 898 females and is thus the largest meta-analysis available in the current literature. The majority of patients had undergone lateral meniscal transplantation (1594 pts). The follow-up time ranges widely from two months to 25 years.

A number of variables have been used in defining proper patient selection criteria. The most commonly used indication was the younger age with pain after meniscectomy not responding to conservative treatment, with normal axial alignment, a stable knee joint and less than Grade 4 cartilage degeneration in the affected compartment. Routinely inflammatory joint diseases, infection and morbid obesity have been excluded. Interestingly, the majority of patients underwent a combined surgery including meniscus allograft transplantation and a wide range of associated procedures (including anterior cruciate ligament reconstruction, osteotomy and cartilage repair [5, 7, 22, 24, 28, 30, 63, 66]). Only 38.8% underwent isolated meniscus allograft transplantation.

Most meniscal allograft transplantations have been performed using arthroscopic-assisted techniques. Very early on, open surgery was performed when dealing with open multiligament repair after trauma [66]. The preferred preservation technique was fresh frozen or cryopreserved in most cases. Lyophilization has been abandoned because of tissue deformation [66].

Regarding meniscal allograft fixation techniques, most studies (37.1%) have been using bone block fixation to obtain better hoop stress retaining biomechanics. In 9.1% the fixation technique was not explicitly described. No correlation could be found between surgical fixation technique and outcome.

A variety of subjective and objective clinical outcome scores have been used: Lysholm in 28.8%, IKDC in 14.2%, VAS in 15.5% and KOOS in 13.6%. All studies describe a significant clinical improvement after transplantation with a tendency to decline with follow-up time [3, 4, 8–19, 21, 22, 24, 26–28, 30–41, 43–55, 57–64]. Survival of MAT has been documented in 54 out of 65 studies. Most frequently, survival was defined as explantation of the graft or conversion to arthroplasty, although some authors used more strict criteria thus resulting in significantly lower survival rates. The mean survival rate in this meta-analysis was 80.9%. Ten per cent of the patients needed conversion to a prosthesis.

Overall, the MAT procedure and the healing rate are comparable to standard arthroscopic meniscal repair surgery. When evaluating the allograft with arthroscopy most authors refer to good integration of the allograft in situ. Some studies refer to shrinking of the allograft [9, 14, 29, 32, 36, 50, 56].

These arthroscopic findings correlate with radiological imaging results (X-ray standing, CT and MRI). X-ray classifications retain an overall decline of osteochondral status of one grade illustrating the absence of long-term chondroprotective effect after meniscal allograft transplantation [13, 15, 34, 37, 39, 42, 46, 47, 51, 54, 63, 64].

Almost 500 allograft menisci were MRI graded. Seventy-five percent presented with an abnormal signal intensity. However, no correlation was observed between the clinical outcome and the presence or absence of an abnormal signal intensity [13, 15, 34, 37, 39, 42, 46, 47, 51, 54, 63, 64].

No correlation could be identified between the mean patient age of the study and the outcome or survival rate. The patient’s gender did not correlate with outcome, although the number of female patients was relatively smaller. There was a slightly minor negative effect on the outcome in BMI > 25 [35, 46]. Concomitant surgery did not influence results negatively when compared with isolated meniscal allograft transplantation. This is possibly due to the fact that these additional surgeries are a clinical requirement and improve weight-bearing chondral properties and function in the index knee.

Time to index surgery from meniscectomy is only illustrated in one study. Jiang et al. [38] compared MAT after meniscectomy and meniscectomy alone and found better subjective results in the former patient group. All studies of this meta-analysis however did not show differences in results when comparing >5 years versus <5 years time delay between meniscectomy and index MAT surgery.

A weakness of the study is the fact that all conclusions were based on reported means. It is possible some of these results are over- or underestimated. Thus further research is definitely needed. Furthermore, studying cost-effectiveness of MAT could be useful since most patients are still part of the economically active population and MAT seems more of a temporary solution for post-meniscectomy pain. Furthermore, it has to be stated that due of no international guidelines some data were too heterogeneous to perform a thorough analysis of it such as the complications, arthroscopic and radiological findings. Therefore these data were compiled and an overall conclusion was stated. Study design did not differ significantly from each other among the analysed studies. However, failure criteria should be more uniform to get a better view of the significant difference in survival rate that this study found. As it is clear that if a certain subjective threshold is used, the results are less favourable. It also needs to be noted that further long-term investigation is needed as there is quite a big incline in failure rate as the follow-up time is longer. There should also be an effort to improve the methodological quality of studying this procedure as most authors are in fact the performing surgeons. Furthermore, data was commonly selected out of a patient database which can also be seen as a flaw in this study, as it possibly consists of data with a possible selection bias.

In conclusion, it appears that MAT is a viable solution for the younger patient with chronic pain in the meniscectomised knee joint. The complications are not severe and comparable to meniscal repair. The overall failure rate at final follow-up is acceptable and the allograft heals well in most cases. However, it tends to have a greater failure rate at longer follow-up. Thus it should be seen as a more temporary solution of post-meniscectomy pain. Progression of osteoarthritis is acceptable at mid-term evaluation. Increased signal on MRI is a common finding without clinical complaints. The correct approach to the chronic painful total meniscectomised knee joint thus requires consideration of all pathologies including alignment, stability, meniscal abnormality and cartilage degeneration. It requires possibly combined but appropriate action in that order. However, published data are of low methodologic quality, thus an effort should be made to improve it and give an opportunity to make more empowered conclusions about the subject.

## Conflict of interest

On behalf of all authors, the corresponding author states that there is no conflict of interest.

## Appendix A

**Table A1.** Overview of all articles.

Author(s)	Graft type	Irradiation	Sizing	Technique	Fixation	Associated procedures
1 Von Lewinski et al., Wirth et al. [64, 66]	16L, 6F HLA matched	L: yes, F; no	No medial or contralateral lateral	Open	D S	3 Microfractures procedures, 1 tractopexy, 23 ACLR, 19 MCL
2 Garrett [33]	16F, 28C	No	Age, weight and afterwards MRI	Open	D S	13 OT (Tibial andfemoral), 11 OAlT, 27 ACLR, 2 OAT, 1 OAT + ACLR
3 Shelton and Dukes [60]	N/A	N/A	RX or CT	Arhtro	B	NA
5 Veltri et al. [63]	F or C	No	RX or MRI	Open/Arthro	B	2 Isolated, 10 ACLR, 1 PCLR, 1 both cruciate ligaments
6 van Arkel et al., van Arkel and de Boer, van der Wal et al. [9–11, 62]	C	No	RX	Open	D S	61 Isolated, 2 ACLR
7 Noyes and Barber-Westin [68]	F	Yes	N/A	Open	B or S (1 hornonly)	N/A
8 Potter et al. [69]	F	No	RX	4 Open/20 Arthro	B	16 ACLR, 1 MLC repair, 1 OT (HTO)
9 Verdonk et al. [3, 6, 7]	V	No	N/A	Open	D S	69 Isolated, 17 OT (15 HTO valgus, 2 DFO varus), 3 ACLR, 4 OAT, 3 microfractures
10 Cameron and Saha [24]	F	Yes	N/A	Open	D S	21 Isolated, 5 ACLR, 34 OT (18 HTO valgus, 10 HTO varus, 6 DFO varus), 7 ACLR & OT (HTO varus)
11 Carter [26]	C	No	RX	Arthro	B	11 Isolated, 30 ACLR, 4 OT (HTO valgus), 1 MCL
12 Kölbel [43]	V	No	RX	Open	S	24 Isolated, 10 OT valgus, 1 ACLR, 1 supracondylar femoral varus OT
13 Stollsteimer et al. [61]	C	No	RX	Arthro	B	22 Isolated
14 Rodeo et al. [52]	F	No	N/A	25 Arthro/3 Open	20 B13 S	8 Isolated, 19 ACLR, 1 OT (HTO)
15 Rath et al. [51]	C	N/A	RX of MRI	Arthro	21 B 1 S	3 Isolated, 11 ACLR (5 revisions, 3 contralateral partial meniscectomies, 1 contralateral meniscal repair, 1 TTT)
16 Ryu et al. [56]	N/A	N/A	N/A	Open	B	12 Isolated, 14 ACLR
17 Yoldas et al. [67]	F	No	N/A	Open	B	11 Isolated, 20 ACLR
18 Felix and Paulos [31]	C	No	RX	Arthro	B	9 Isolated, 18 ACLR, 2 OT, 4 ACL & OT
19 Sekiya et al. [58]	C	No	RX	Arthro	B	19 ACL, 9 revision ACL
20 Noyes et al. [12]	C	No	RX	Arthro	B	16 OAT, 6 ACLR, 1 ACLR & MCL, 1 PCLR, 1 both cruciate ligaments
21 Fukushima et al. [32]	C	No	RX	Open	T	8 ACLR, 1 OT (HTO)
22 Graf et al. [35]	C	Yes (except 1)	RX	Open	7 B1 S	3 ACLR
23 Cole et al. [29]	C, <20% F	No	RX	Open	B	21 Isolated, 3 osteochondral allografts, 3 OAT, 2 microfractures, 2 OCD fixations, 1 ACI, and 1 chondral debridement, 1 OT, 6 ligament reconstructions and 1 OT (HTO valgus)
24 Sekiya et al. [59]	C	No	RX	Arthro	17 B8 T	25 Isolated
25 Stone et al. [18]	18 F, 29 C	No	N/A	Open	T	7 Isolated, 13 patients 1, 24 patients 2 and 1 patient 3 associated procedure(s), 6 ACLR, 14OT (HTO valgus), 19 microfractures, 47 femoral condyle chondroplasty, 24 articular cartilage paste resurfacing
26 Rankin et al. [50]	C	No	RX	Open	B	2 Isolated, 4 ACLR, 4 OAT
27 Rueff et al. [55]	C	No	RX	Arthro	B	8 Isolated
28 Kim and Bin [14]	2 C, 12 F	No	RX	Arthro	B	3 OAT
29 Hommen et al. [37]	C	No	RX	Open	6 B13 D S1 mixed	5 Isolated, 9 ACLR, 1 revision ACL, 2 OT (HTO 1 varus, 1 valgus), 3 loosened retinacula, 3 adhesiolysis, 1 loose body, 2 capsular plication, 3 femoral condyle chondroplasty
30 Farr et al. [30]	F	No	RX	Arthro	B	29 ACI, 16 patients: 6 OT (HTO valgus), 3 TTT, 8 ACL
31 Bhosale et al. [23]	C	No	N/A	Open	B	8 ACI
32 Lee et al. [45]	F	No	RX	Arthro	B	17 Isolated, 4 ACLR
33 Chang et al. [27]	C	No	RX	Arthro	NA	8 Isolated, 2 ACLR, 1 ACL revision, 1 MCL reconstruction
34 Rue et al. [54]	C or F	N/A	N/A	Open	B	All patients chondral repair (16 ACI, 15 OAlT), 2 hardware removal, 1 OT (HTO)
35 Lee et al. [46]	F	No	RX	Arthro	B	NA
36 Gomoll et al. [70]	F	No	RX	Arthro	B	7 Chondral repair (5 OAlT, 3 microfractures, 1 ACLR, 1 OAT & OT, 5 HTO valgus, 2 DFO varus)
37 LaPrade et al. [44]	F	No	High field MRI	Arthro	D SB	19 Isolated, 6 ACLR, 4 ACL revision, 4 hardware removal, 5 microfractures, 3 osteoarticular allografts, 3 distal femoral osteotomies
38 Kim et al. [15]	C or F	N/A	RX	Arthro	D SB	82 Isolated, 22 ACLR, 4 OCD repair, 1 ACLR & posterolateral horn repair, 1 PCLR
39 Kim et al. [41]	C or F	N/A	RX	Arthro	D SB	N/A
40 Abat et al. [21]	F	No	RX	Arthro	33 S55 B	18 ACLR, 15 microfractures, 9 chondral debridement, 3 hardware removal, 2 arthroscopic chondral repair with TruFit plugs
41 Cole et al. [28]	F	N/A	RX	Arthro	D SB	8 Isolated, 5 ACLR, 3 ACL revision, 2 microfractures, 2 OAlT, 3 hardware removal, 1 PC thermal shrinkage
42 Kazi et al. [39]	F	N/A	N/A	Arthro	S	53 Osteotomies, 7 ACLR
43 Marcacci et al. [47]	F	No	RX en MRI	Arthro	D SB	22 Isolated, 4 ACLR (3 autografts, 1 allograft), 6 osteotomies (3 HTO, 3 DFO)
44 González-Lucena et al. [34]	F	No	RX	Arthro	S	8 ACLR, 8 microfractures, 9 chondral debridement
45 Ha et al. [36]	F	N/A	RX	Arthro	B	15 ACLR, 2 PCLR, 4 posterolateral twisting instability, 2 osteotomies
46 Stone et al. [19]	F (94) or C (24)	Yes (1 case)	RX	Arthro	B	67 Articular cartilage enting 69 microfractures, 15 Medial opened tibial OT, 17 ACLR (10 bone-patellar tendon allografts, 6 middle third patellar tendon autografts, 1 Achilles tendon allograft)
47 Vundelinckx et al. [4, 5]	C	N/A	CT	Arthro	2 B33 S	2 Microfractures
48 Alentorn-Geli et al. [22]	F	N/A	RX and MRI	Arthro	D S B	N/A
49 Jiang et al.[38]	C	N/A	RX, CT and MRI	Arthro	B S	6 ACLR, 1 meniscus repair, 1 partial meniscectomy
50 Saltzman et al. [57]	F	No	RX	Arthro	B	8 Isolated, 5 ACLR, 3 ACL revision, 2 microfractures, 2 OAT, 2 OAlT, 3 hardware removal, 1 PC thermal shrinkage
51 Kempshall et al. [40]	N/A	N/A	RX, MRI and arthro	Arthro	T	13 DFO, 8 HTO, 14 ACRL, 1 meniscus repair, 7 MACI femur, 3 MACI tibia, 1 MACI trochlea, 16 microfractures tibia, 15 microfractures femur, 2 TruFit plugs
52 McCormick et al. [48]	F	No	RX	Arthro	B	81 Isolated MAT, 74 chondral procedures, 14 OT and chondral procedures, 23 ACLR, 8 OT
53 Roumazeille et al. [53]	F	No	RX	Arthro	S	5 ACLR
54 Campbell et al. [25]	F	No	RX	Arthro	B	10 ACI, 2 ACLR, 1 ACI biopsy, 1 OAT, 3 OAlT, 1 HTO
55 Kocher et al. [42]	N/A	No	RX	Arthro	B	1 Chondroplasty, 1 adhesiolysis
56 Noyes and Barber-Westin [13]	C	No	RX and MRI	Arthro	B	20 OAT, 6ACI, 2 ACLR
57 Waterman et al. [65]	N/A	N/A	N/A	Arthro	N/A	13 HTO, 24 chondral procedures, 3 PCLR, 7 other
58 Zaffagnini et al. [16]	F	No	RX	Arthro	T	12 HTO, 1 HTO + ACL, 1 HTO + osteochondral scaffolding, 2 DFO, 9 ACLR, 2 ACL revision, 2 mosaicplasty, 3 osteochondral scaffolds, 9 microfractures
60 Zaffagnini et al. [17]	F	No	RX	Arthro	T	23 HTO, 4 HTO + ACL, 2 HTO + osteochondral scaffolding, 4 DFO, 17 ACLR, 1 PCL, 2 ACL revision, 3 mosaicplasty, 3 osteochondral scaffolds, 11 microfractures
61 Van Der Straeten et al. [8]	FV	No	N/A	Open, arthro	N/A	50 Microfractures, 2 OAT, 39 HTO, 27 ACLR

L: lyophilized, F: fresh-frozen, C: cryopreservation, V: viable, HLA: human leukocyte antigen, Arthro: arthroscopically, S: soft tissue fixation, D S: direct fixation to the capsule, T: transosseous fixation, B: bony fixation, ACLR: anterior cruciate ligament reconstruction, MCL: medial collateral ligament repair, N/A: nonavailable data, OT: osteotomy, HTO: high tibial osteotomy, DFO: distal femoral osteotomy, PCLR: posterior cruciate ligament reconstruction, TTT: tibial tuberositas transfer, OAT: osteochondral autograft transfer, OCD: osteochondritis dissecans treatment, ACI: autologous chondrocyte implantation, MACI: matrix induced autologous chondrocyte implantation, OAlT: osteochondral allograft transfer, PC: posterior cruciate.

## Appendix B

**Table B1.** Associated procedures.

Procedure	*N*	%
Microfractures	224	8.2
Tractopexy	1	0.0
Osteotomy	345	12.6
OAlT	39	1.4
OAT	59	2.2
ACLR	536	19.5
ACL revision	17	0.6
PCLR	9	0.3
MLC	4	0.1
Contralateral meniscectomy/repair	6	0.2
ACL + PCL	2	0.1
OCD	6	0.2
ACI	81	3.0
Chondral debridement	19	0.7
Hardware removal	15	0.5
Chondroplasty	98	3.6
Paste resurfacing	91	3.3
Trufit	4	0.1
Ligament reconstruction	6	0.2
Retinaculum release	3	0.1
Adhesiolysis	3	0.1
Removal of loose tissue	1	0.0
Capsular plication	2	0.1
TTT	4	0.1
Osteoarticular allografts	3	0.1
PC thermal shrinkage	2	0.1
Posterolateral turning instability	4	0.1
Nondefined chondral procedures	83	3.0
MACI	10	0.4
Isolated	1065	38.8
Total amount of procedures	2742	100

OAlT: osteochondral allograft transfer, OAT: osteochondral autograft transfer, ACLR: anterior cruciate ligament reconstruction, PCLR: posterior cruciate ligament reconstruction, MCL: medial collateral ligament repair, OCD: osteochondritis dissecans treatment, ACI: autologous chondrocyte implantation, TTT: tibial tuberositas transfer, PC: posterior cruciate, MACI: matrix induced autologous chondrocyte implantation.

## Appendix C

**Table C1.** Reported complications.

Reported complications	*N*	%
Microfractures	1	0.2
Anterior cruciate ligament reconstruction	1	0.2
Venous thrombo-embolism	3	0.5
Compartment syndrome	1	0.2
Hardware removal	24	3.6
Patella tendon repair	2	0.3
Matrix induced autologous chondrocyte implantation	1	0.2
Algoneurodystrophy	2	0.3
Meniscal tear	214	32.3
Arthrofibrosis	39	5.9
Loosening	32	4.8
Debris	138	20.8
Need for mobilization	14	2.1
Granuloma	6	0.9
Adhesions	2	0.3
Chondroplasty	42	6.3
Loose body removal	13	2.0
Osteophytosis	12	1.8
Baker cyst	2	0.3
Synovitis	15	2.3
Pain	14	2.1
Osteotomy	19	2.9
Loose plug	2	0.3
Phlebitis	1	0.2
Pulmonary embolism	2	0.3
Infection	28	4.2
Hemarthrosis	3	0.5
Neuropraxia	4	0.6
Effusion of the allograft	8	1.2
Hepatitis C	1	0.2
Notchplasty	17	2.6
Total	663	100
